# Perspectives of an NCI T32 Training Program Designed to Train a Diverse Workforce in Cancer Health Equity Research

**DOI:** 10.1007/s13187-024-02514-w

**Published:** 2024-10-04

**Authors:** Mackenzie A. Ferguson, Lisa Tussing Humphreys, Ifeanyi Beverly Chukwudozie, Margaret E. Wright, Caryn E. Peterson, Marian L. Fitzgibbon, Andrew McLeod

**Affiliations:** 1 Department of Pediatrics, University of Illinois Chicago, Chicago, IL USA; 2 University of Illinois Cancer Center, Chicago, IL USA; 3 Department of Kinesiology and Nutrition, University of Illinois Chicago, Chicago, IL USA; 4 Data Integration Shared Resource, University of Illinois Cancer Center, Chicago, IL USA; 5 Department of Epidemiology & Biostatistics, University of Illinois Chicago, Chicago, IL USA; 6 Institute for Health Research and Policy, University of Illinois Chicago, Chicago, IL USA

**Keywords:** Cancer Education, Cancer Prevention, Cancer Control, Cancer Research Training, Training Grant, Postdoctoral Training, Predoctoral Training

## Abstract

With cancer health disparities on the rise in the United States (USA), there is an increased need for novel approaches to address these challenges. One such approach that may help address these disparities is increasing diversity in the biomedical research workforce. The Cancer Health Equity and Career Development Program (CHECDP) embodies this approach by recruiting and training underrepresented minorities in cancer research to develop the skills and training needed to be competitive for independent research careers, thus diversifying the biomedical research workforce. The training model that CHECDP employs is unique with its funding through the NCI training mechanism, its strong institutional support, and its participant-driven curriculum. The curriculum includes educational, career, and leadership opportunities that are continuously evaluated for sustained impact. The program has been comprised of mostly under-represented minorities that have been propelled to independent careers with a high rate of funded career development awards. Our T32 program serves as a model of success for other programs seeking to diversify the biomedical research workforce and reduce cancer health disparities.

## Introduction

In the USA, cancer is the second leading cause of death. Data from the American Cancer Society report that in 2024 there will be an estimated 2 million new cancer cases and 612,000 deaths [1]. The USA is a racial and ethnically diverse nation, benefiting from cultures and perspectives that diversity brings, but this also presents challenges. Although cancer mortality rates have been declining, underrepresented minority (URM) groups continue to bear a disproportionate burden of disease. For example, Black Americans have 13% higher overall cancer mortality rates than non-Hispanic whites, and this disparity is even more pronounced for cancers of the prostate (107% increase), breast (40% increase), and colon and rectum (31% increase) [2]. As another example, though Hispanics generally have lower cancer mortality rates than non-Hispanic whites, they bear 48% and 130% higher mortality from liver and stomach cancers, respectively. As the proportion of minority individuals in the USA grows, these inequities present an increasing public health concern [3].

For example, one strategy to overcome these inequities is to increase diversity in the biomedical workforce [4]. While increasing workforce diversity is recognized as a partial solution, URM researchers continue to remain less represented at each career stage [5, 6]. Increasing the number of diverse investigators provided with opportunities for mentored research training and career and leadership development fosters independence critical to increasing capacity in cancer health equity research.

The need to diversify the training opportunities for URMs has resulted in the development of relevant federal training programs to enhance diversity [7]. Grounded in our extensive experience with education, mentored research training, career and leadership development, and a commitment to diversifying the biomedical workforce, the University of Illinois Chicago (UIC) Cancer Health Equity and Career Development Program (CHECDP) focuses on the transdisciplinary skills needed to address cancer inequities. UIC is uniquely positioned to host this training program. UIC is located in the third largest USA city, is a minority serving institution (MSI), and has one of the most diverse student bodies in the nation. UIC also has vast resources and partnerships, including the University of Illinois Cancer Center (Cancer Center), UI Health, and the 11-site Mile Square Health Center Federally Qualified Health Center (FQHCs)—the oldest FQHC in Illinois. These attributes position UIC as a leader in community-engaged cancer research, referred to as the “Community to Bench Model” of research [8–10]. Funded through NCI initially as an R25T in 1992 and then transitioning to a T32 in 2018, the CHECDP trains three predoctoral fellows and three postdoctoral fellows each year.

There are three primary reasons to focus on expanding and improving pre and postdoctoral training programs in cancer health equity with a core emphasis on training URMs. First, conducting cancer health equity research faces unique challenges. The challenges result from studying historically underrepresented/underserved populations, which requires specialized training in cancer behavioral risk factors, social determinants of health, community outreach, research design, measurement, and outcome assessment [11, 12]. Second, it is critical to include URMs as members of cancer disparities research teams due to their lived experiences and better understanding of the needs of their communities. This includes a deeper comprehension of risk and protective behaviors and barriers to medical care [13]. Black American and Hispanic adults have less access to resources that promote a healthy lifestyle, including green space, grocery stores, and satisfactory air quality. As a result, they experience disparities in cancer risk behaviors and have a greater incidence of physical inactivity and obesity, less healthful diets, and increased challenges to quitting tobacco use compared to non-Hispanic white adults [14]. Third, cancer health equity research is an interdisciplinary field requiring training programs that are inclusive of diverse investigators and disciplines. We offer this article as a model of how a program can effectively address these training needs.

## Comprehensive Training

### Expectations

In the initial year, all fellows select a Transdisciplinary Mentoring Team (TMT). Fellows work to develop a Transdisciplinary Individual Development Plan (TIDP) to create benchmarks for their progress. Through the TIDP, fellows inventory their current skills and areas for improvement, setting short and long-term goals with detailed action steps. Fellows are expected to submit publications in each year of the fellowship, and mentors are encouraged to support fellows as first author on at least one paper from the mentored research project. Fellows are also expected to present first-authored conference presentations in each year of the program. The postdoctoral fellows must additionally complete and submit a grant proposal (e.g., F32, R21, and K award) before the end of their program. During their first year, pre-doctoral fellows’ complete coursework relevant to the dissertation and are encouraged to write an F31 application. By the end of year one, pre-doctoral fellows defend their dissertation proposal and are expected to complete the dissertation by the end of year three. Progress towards these goals is assessed when fellows and mentors meet with the CHECDP Co-Directors semiannually to review their TIDP.

### Education

The CHECDP’s educational goals are met through academic coursework, core curriculum (through weekly seminars), and other scientific research training opportunities as identified by each fellow’s needs. Course requirements depend on the type of fellow and their career goals, research expectations, and areas of need.

A typical pre-doctoral course plan encompasses both departmental requirements and CHECDP requirements. Divisional requirements include traditional coursework in research and statistical methods, systems and policy courses, and topics in health as related to their concentration. Non-credit requirements for the CHECDP include human subjects CITI and HIPPA training and additional training as identified from their TIDP.

Postdoctoral fellows have PhD or MD degrees, and while most have prior research experience, some MDs may not and require additional training in research methodologies. At the start of the program and at each semiannual review, fellows and mentors discuss needed coursework to address any gaps. Common selections include the Health Disparities Certificate Program, courses in qualitative methodology, advanced biostatistics, and social epidemiology. In many cases, postdoctoral fellows can complete coursework online and in less than a year.

## Career and Leadership Development

### Structured Career and Leadership Development

Structured career development training is provided via the Core Curriculum for all fellows in weekly CHECDP seminars. These seminars provide opportunities for education, skill building, near-peer mentorship, scientific writing support, and exposure to cancer and health equity-related disciplines, clinicians, and scientists. Table 1 presents an overview of the core curriculum from 2021 to 2022 and 2022 to 2023.

**Table 1:** CHECDP core curriculum, 2021–2022 and 2022–2023 academic years

	2021–22 academic year	2022–23 academic year
	Core curriculum: grantsmanship and research dissemination	Core curriculum: developing your professional portfolio
Session topic	“Grant Development I: Identifying Opportunities”	“The Professional Scientific Portfolio”
	“Working with the IRB”	“Drafting your DEI Statement”
	Grant Development II: Budget Development	“Drafting your Teaching Statement”
	“Developing your Research Aims”	“The NIH Biosketch”
	“Data Visualization: Presenting your data with Tableau”	“Best Practices from Successful Career Development Award Applicants” T32 Postdoctoral Alumni
	“Policy Briefs: from Research to Advocacy”	“Job-Search Lessons Learned Panel” T32 Predoctoral Alumni
Session topic	Mini-topic: Career Development	Mini-topic: Leadership & Communication
	“Developing your IDP”	“FIRO-B: Leadership Assessment”
	“Mentoring Best Practices”	“Managing Up”
	“Interviewing Skills”	“Prioritizing”

### Fellow-Driven Career and Leadership Development

With a focus on the individual needs of our fellows, the CHECDP T32 encourages weekly mentoring meetings, formal career coaching, mini-topics chosen by the fellows (e.g., prioritizing (see Table 1)), and attendance at conferences relevant to our fellows’ unique research goals. Each semester’s schedule also includes one or two core topics tailored to the needs of the group at that time.

### Peer/Near-Peer Mentoring

Each summer, fellows have an opportunity to participate in near-peer mentoring of high school and undergraduate students through the Diversity in Cancer Research (DICR), Cancer Health Equity Summer Scholars (CHESS), and ResearcHStart programs at the Cancer Center, and the NCI funded U54 CHEC Summer Scholars program. The mentees are matched with fellows who are interested in similar research areas. Fellows provide guidance in human subjects’ research, literature searches, scientific writing, data analysis, and shadowed research experiences, culminating in a poster presentation at a research symposium.

### Work-in-Progress Sessions

At least once each semester, fellows share their work (e.g., a scientific paper or conference presentation) for peer review. Using skills gained in peer-to-peer mentoring sessions, fellows provide formal feedback to their peers. The breadth of fellows’ projects allows for exposure to research across the cancer continuum.

### Additional Career and Leadership Development Opportunities

Additional career-focused seminar topics include the following:

- Professional development. We incorporate sessions on professional development, including networking, creating an elevator speech, working effectively with mentors, and career planning. Guest speakers include former fellows who discuss their current positions and their postdoctoral/job search strategies.
- Professional portfolio. In preparation for their next career step, fellows develop a Professional Portfolio that includes their CV, NIH biosketch, NCBI bibliography, research and teaching statements, and personal statements. These materials are developed and reviewed in workshops led by external experts.
- Writing accountability and summer writing groups. Throughout the academic year, fellows participate in peer-to-peer writing accountability groups. Although there is no structured curriculum, we use the summer term to address fellow-specific needs, such as writing support, peer review of papers in progress, and completion of other training.

### Proposal Development and “Mock” Grant Review

Each postdoctoral fellow is encouraged to take an Advanced Writing Course to support them as they develop a required grant proposal (typically a K career development award, an R03, or an R21). The course takes fellows through the application components and culminates in a mock grant review. Internal reviewers who have familiarity with the NIH review process participate from a broad range of UIC disciplines and departments. Reviewers provide feedback guided by the NIH scoring criteria and an overall impact summary. Fellows also receive detailed written reviews to aide with further revisions to their proposal.

### Formal Career Coaching

Each fellow attends two to three group sessions per year with a professional career coach. This opportunity is provided through the Sparks Group, which is an organization that offers one-on-one and small group coaching to maximize leadership development. Through this program, fellows have an opportunity to dive deeper into topics through individualized coaching. These professional coaching sessions are designed to address specific areas that fellows want to address (e.g., self-promotion, presentation skills, confidence, limit setting, and goal setting) [15, 16].

### (SBM) Diversity Institute for Emerging Leaders

Postdoctoral fellows are sponsored by the Cancer Center to apply to and attend the Society of Behavioral Medicine (SBM) Diversity Institute for Emerging Leaders. This institute helps early career researchers from diverse backgrounds or those working with underrepresented groups develop into leaders by fostering a deeper understanding of the principles of diversity and inclusion. The program begins with a one-day intensive workshop at the SBM annual meeting and continues through the year with quarterly learning opportunities and regular small group sessions with peers and mentors.

### Fundamental Interpersonal Relationship Orientation-Behavior™ (FIRO-B)

To strengthen communication and leadership abilities, fellows participate in a FIRO-B workshop. The FIRO-B test is used to understand how you work in teams. The instrument is based on the theory that people’s behavior is motivated by their interpersonal needs, which are grouped in 3 categories: inclusion, control, and affection. Fellows use personalized results to engage in 1 on 1 sessions with a coach to understand how they can best communicate and work efficiently in teams.

## General Candidate Identification, Qualification, and Application Process

### Prospective Predoctoral Candidates

Predoctoral fellows are selected from outstanding PhD students interested in independent cancer research careers. We work with faculty to identify potential candidates enrolled in cancer research programs in the UIC health science colleges, College of Arts and Sciences, or Engineering.

### Prospective Postdoctoral Candidates

Postdoctoral candidates should have an appropriate scientific background in basic, clinical, behavioral, or social science with documented research training (apart from MDs as noted below).

### Additional Considerations for Medical Postdoctoral or Other Clinically Trained Fellows

Potential fellows with a clinical background may require additional didactic courses beyond the supplemental courses and certificate programs required of most postdoctoral fellows. Because of the unique perspective that MDs bring to the training program, the CHECDP is eager to recruit MD fellows.

### Application

Applicants submit (1) a CV, (2) three letters of recommendation, (3) three writing samples, and (4) an application letter noting their areas of interest in cancer control and health equity that outline their career objectives.

### Recruitment Plan to Enhance Diversity

UIC offers a rich environment for the T32, with access to expertise in 34 departments and 8 colleges across the university. Recognizing the importance of creating a diverse workforce, we use several strategies to attract underrepresented pre- and postdoctoral fellows to UIC including (1) encouraging mentors to promote our programs at national meetings and highlighting our interest in recruitment of URM fellows, (2) maintaining communication with our former fellows to provide information to potential applicants, (3) requesting colleagues that direct training programs focused on cancer and health equity to nominate potential candidate to apply, and (4) expanding our advertising for fellows openings to include the minority caucuses of American Public Health Association (APHA) and the American Psychological Association (APA) and other health professional organizations.

## Fellows and Outcomes

### Evaluation Plan

We continuously assess program impact according to the proposed aims with review by the leadership team and the Internal and External Advisory Committees (Table 2). These reviews help to implement a cohesive model of process- and outcome-based evaluation using a data-driven approach to track, monitor, and evaluate program activities and goals, fellows’ progress, and mentor–mentee relationships to enable us to adapt activities as needed process evaluation assesses the implementation of the training plan (TIDP) activities to ensure all fellows are supported in education, mentored research, and career and leadership development. The primary goal is to provide the didactic and transdisciplinary research opportunities, skill development, mentoring, and resources necessary to become independent researchers focused on cancer health equity. Longer-term criteria for success of the training program include (1) fellow success in obtaining an academic position where they can pursue cancer health equity research; (2) fellow contributions to the field of health disparities/health equity as evidenced by the publication of peer-reviewed articles, book chapters, and scientific presentations; and (3) fellow success in obtaining NCI external funding for original research. The program’s success and impact in achieving the proposed aims are evaluated annually by the leadership team, IAC, and EAC.

**Table 2:** Summary of CHECDP T32 over the past 10 years (2013–2023)

**Predoctoral fellows outcomes** (*N* = 16)			
Status of predoctoral fellows	3 current; 13 completed		
Accepted postdoctoral or academic positions	14 of 16 (87.5%)		
Status of grant proposals	Grant type	Submitted	Awarded
	F31	2	1 (50%)
	Diversity supplement	1	1 (100%)
Underrepresented minorities	10 of 16 (62.5%)		
**Postdoctoral fellow outcomes** (*N* = 16)			
Status of postdoctoral fellows	3 current; 13 completed		
Accepted assistant professor or equivalent positions	11 of 16 (68.75%)		
Status of grant proposals	Grant type	Submitted	Awarded
	Career development grant (e.g., K99)	9	7 (77%)
	R34	1	1 (100%)
Underrepresented minorities	8 of 16 (50%)		
**Publications** (*N* = 405)			
First authored publications	99 of 405 (24.44%)		
Collaborative publications between fellows	46 of 405 (11.36%)		

We track CHECDP alumni as they progress on their career paths. For MD fellows who complete our program and then proceed with additional specialty fellowships, the attainment of these goals may be somewhat delayed, but annual contacts will be made, and progress will be documented.

#### Exit Interviews

When each fellow completes the program, they complete an exit survey reflecting on their time as a fellow and the strengths and weaknesses of the CHECDP. This feedback is used for continual improvements. Top highlights of the program often cited by fellows include a large amount of protected time and available resources that facilitate attendance at conferences, publishing papers, and participation in a variety of other career development activities. Some recent suggestions for improvement include having certain seminar topics be for postdoctoral fellows or predoctoral fellows only (to match skill level) and for leadership to push fellows to set more challenging goals at biannual reviews.

## Conclusions

Given the well-documented cancer health disparities in the US, cancer health equity training programs are uniquely positioned to improve equity in cancer outcomes. The CHECDP provides a unique approach by focusing on the development of training a diverse workforce. The training of the CHECDP is focused on curriculum-based education, mentored research training, and career and leadership development. The CHECDP represents a replicable model to increase diversity in pre and postdoctoral cancer health equity training.
